# Uncommon Development of Intra-abdominal Abscesses Following Acute Pancreatitis: A Case Report

**DOI:** 10.7759/cureus.98798

**Published:** 2025-12-09

**Authors:** Ian Engerbretson, Marina Botros, Steven J Laxton

**Affiliations:** 1 Department of Emergency Medicine, UTHSC (The University of Tennessee Health Science Center) Nashville - St Thomas Health, Murfreesboro, USA; 2 Department of Emergency Medicine, UTSHC (The University of Tennessee Health Science Center) Nashville - St Thomas Health, Murfreesboro, USA

**Keywords:** abdominal pain, acute pancreatitis, intra-abdominal abscess, pancreatitis, perinephric abscess

## Abstract

Acute pancreatitis is not an uncommon gastrointestinal condition with the potential for both local and systemic complications. While pancreatic necrosis and pseudocyst formation are well-recognized complications, the formation of distant intra-abdominal abscesses is a rare occurrence. We report the case of a 52-year-old man who presented to the emergency department with abdominal pain, noted to be secondary to pancreatitis. Abdominal imaging additionally revealed a left perinephric abscess with additional intra-abdominal abscesses thought to be secondary to acute pancreatitis.

This case highlights an uncommon complication of pancreatitis and underscores the importance of considering extra-pancreatic abscess formation in patients with persistent or recurrent abdominal symptoms.

## Introduction

The pancreas is a glandular organ located behind the stomach in the upper abdomen. It plays a vital role in both the digestive (exocrine) and endocrine systems. Structurally, it is about 6 inches long and shaped somewhat like a flat pear. The pancreas is composed of two main parts: the exocrine tissue, which produces digestive enzymes, and the endocrine tissue, which produces hormones such as insulin and glucagon [1].

The pancreas has two primary functions: aiding digestion and regulating blood sugar levels. Digestive enzymes help break down fats, proteins, and carbohydrates in the small intestine, while hormones like insulin and glucagon maintain glucose homeostasis. Together, these functions are essential for nutrient absorption and energy regulation [1,2].

Pancreatitis is an inflammation of the pancreas that can be acute or chronic. It results from premature activation of pancreatic enzymes, leading to autodigestion and tissue injury. Symptoms often include severe abdominal pain, nausea, vomiting, and fever. Pancreatitis can vary in severity, sometimes requiring hospitalization and specialized treatment [3,4].

The most common causes of pancreatitis include gallstones and chronic alcohol use. Gallstones can block the pancreatic ducts, leading to inflammation and damage. Other causes include certain medications, high triglyceride levels, infections, and trauma to the pancreas. In some cases, such as idiopathic pancreatitis, the exact cause remains unknown [2,5].

Pancreatitis can lead to a wide range of complications if not properly managed. Common sequelae include the formation of pancreatic pseudocysts, necrosis, and infection, which may cause permanent loss of pancreatic function and result in endocrine or exocrine insufficiency [6,7]. Chronic pancreatitis, in particular, can lead to malnutrition due to impaired digestion and diabetes from damage to insulin-producing cells. In severe cases, pancreatitis may trigger a systemic inflammatory response with multi-organ dysfunction, which can be life-threatening [4]. Less frequent, but important, complications have also been reported, such as emphysematous pancreatitis [8], pancreatic fistulae [9], portal venous thrombosis [10], pancreatic ascites [11], and ectopic or hepatic pseudocysts [12].

In rare cases, because the pancreas lies in the retroperitoneum, enzyme-rich fluid may also track into unusual spaces. Only a few case reports describe involvement of the renal or perinephric areas. One case report showed a pancreatic pseudocyst imitating a left kidney abscess, similar to what is described in this case [13]. An additional report described a perirenal pseudocyst causing Page-kidney physiology [14]. Perinephric abscess resulting from a pancreatic fistula in chronic pancreatitis has also previously been described [15]. A prior report, similar to the one presented here, documented a retroperitoneal abscess extending into the femoral region as a rare complication of necrotizing pancreatitis [16]. Another report confirmed that renal and perirenal space involvement can occur as part of the extra-pancreatic spread of acute pancreatitis in a dedicated MRI study [17].

These reports highlight an uncommon but clinically important phenomenon of pancreatic infections extending into the perirenal space. Our case is notable because the patient had recently undergone drainage of a left perinephric abscess that cultured without growth and was considered a "sterile" abscess at an outside facility. He then presented with recurrence that was ultimately attributed to acute pancreatitis, likely describing a similar phenomenon to the reports listed above that showed extension of pancreatic fluid into the perirenal space secondary to acute pancreatitis. By describing this case, we aim to increase awareness of perinephric and intra-abdominal abscesses as rare sequelae of pancreatitis to aid clinicians in earlier recognition and appropriate management.

## Case presentation

A 52-year-old man presented to the emergency department at Ascension Saint Thomas Rutherford complaining of generalized abdominal pain. This patient had a past medical history of alcoholic pancreatitis, a remote history of a right lower-extremity deep vein thrombosis resulting in a right above-the-knee amputation (AKA), and drainage of a sterile left perinephric abscess with interventional radiology at an outside facility one week ago. 

The patient was discharged after the drainage of his left perinephric abscess one week prior to arrival without home antibiotics. He was doing well until the day prior to this presentation, when he developed generalized abdominal pain that radiated to his back with associated nausea. He stated that eating made his pain worse. The patient quit drinking alcohol five months ago after being first diagnosed with his first episode of pancreatitis.

Upon presentation to the emergency department, the patient’s vitals were a temperature of 98.1F, heart rate 81 beats per minute, blood pressure 141/75, and 98% oxygen saturation on room air. 

Physical examination revealed the patient to be ill-appearing and uncomfortable. His abdomen was soft and non-distended, with only epigastric and periumbilical tenderness to palpation, with voluntary guarding present. Otherwise, the remainder of the physical examination was unremarkable.

Laboratory studies are shown in Tables 1, 2, 3 for complete blood count, urinalysis, and complete metabolic panel, respectively. Most notable among the laboratory studies were findings of a significant elevation of lipase level concerning for pancreatitis and a leukocytosis with neutrophilic predominance. Blood cultures were obtained, which resulted negative after five days. 

**Table 1:** Complete Blood Count With Differential This table lists the results for the complete blood count with differential with reference range for normal and indicated H (high) or L (low) for abnormal test result. WBC, white blood cell count; RBC, red blood cell count; MCV, mean corpuscular volume; MCH, mean corpuscular hemoglobin; MCHC, mean corpuscular hemoglobin concentration; RDW, red cell distribution width; H, high; L, low.

Test	Results	Normal range	Interpretation
WBC (×10³/mm³)	18.2	4.0-10.5	H
RBC (×10⁶/mm³)	4.38	4.2-5.4	-
Hemoglobin (g/dL)	13.4	13.5-17.5 (M)/12.0-16.0 (F)	L
Hematocrit (%)	39.7	41-53 (M)/36-46 (F)	L
MCV (fL)	90.6	80-100	-
MCH (pg)	30.6	27-33	-
MCHC (g/dL)	33.8	32-36	-
RDW (%)	12.8	11.5-14.5	-
Platelets (×10³/mm³)	700	150-450	H
Neutrophils (%)	84	40-75	H
Lymphocytes (%)	8	20-45	L
Monocytes (%)	6	2-10	-
Eosinophils (%)	1	0-6	-
Basophils (%)	0	0-1	-
Immature Granulocytes (%)	1.4	0.0-0.5	H
Absolute Neutrophil Count (×10³/mm³)	15.2	1.8-7.7	H
Absolute Lymphocytes (×10³/mm³)	1.4	1.0-4.8	-
Absolute Monocytes (×10³/mm³)	1.1	0.1-1.0	-
Absolute Eosinophils (×10³/mm³)	0.2	0.0-0.5	-
Absolute Basophils (×10³/mm³)	0	0.0-0.2	-
Absolute Immature Grans (×10³/mm³)	0.26	0.00-0.04	-

**Table 2:** Results for Urinalysis This table shows the results for urinalysis with reference range for normal if available and indicated H (high) or L (low) or abnormal for abnormal results. WBC, white blood cells; RBCs, red blood cells; H, high; L, low.

Test	Result	Normal range	Interpretation
Color	Yellow	Yellow	-
Appearance	Clear	Clear	-
Specific Gravity	1.049	1.005-1.030	H
pH	6	4.5-8.0	-
Glucose	Negative	Negative	-
Ketones	Negative	Negative	-
Blood	Negative	Negative	-
Protein	Negative	Negative	-
Bilirubin	1+	Negative	Abnormal
Nitrite	Negative	Negative	-
Urobilinogen (EU/dL)	0.2	0.2-1.0	-
Leukocyte Esterase	Negative	Negative	-
WBCs (/HPF)	5	0-5	-
RBCs (/HPF)	0	0-3	-
Casts	None Seen	None	-
Epithelial Cells (/HPF)	0	0-10	-

**Table 3:** Complete Metabolic Panel This table shows the results for the complete metabolic panel including lipase with units, reference range, and indicated H (high) or L (low) for abnormal results. CO₂, bicarbonate; BUN, blood urea nitrogen; AST, aspartate transaminase; ALT, alanine transaminase; eGFR, estimated glomerular filtration rate; H, high; L, low.

Test	Results	Normal range	Interpretation
Sodium (mmol/L)	136	135-145	-
Potassium (mmol/L)	3.8	3.5-5.1	-
Chloride (mmol/L)	108	98-107	H
CO₂ (mmol/L)	18	22-29	L
BUN (mg/dL)	13	7-20	-
Creatinine (mg/dL)	0.7	0.6-1.3	-
eGFR (mL/min/1.73 m²)	109	≥90	-
Glucose (mg/dL)	141	70-99 (fasting)	H
Calcium (mg/dL)	9.6	8.5-10.2	-
Total Protein (g/dL)	7.6	6.4-8.3	-
Albumin (g/dL)	3.7	3.5-5.0	-
Total Bilirubin (mg/dL)	0.3	0.1-1.2	-
Alkaline Phosphatase (U/L)	78	44-147	-
AST (U/L)	12	10-40	-
ALT (U/L)	15	7-56	-
Anion Gap (mmol/L)	10	8-16	-
Lactic Acid (mmol/L)	0.7	0.5-2.2	-
Lipase (U/L)	1090.00	20-80	H

**Figure 1:**
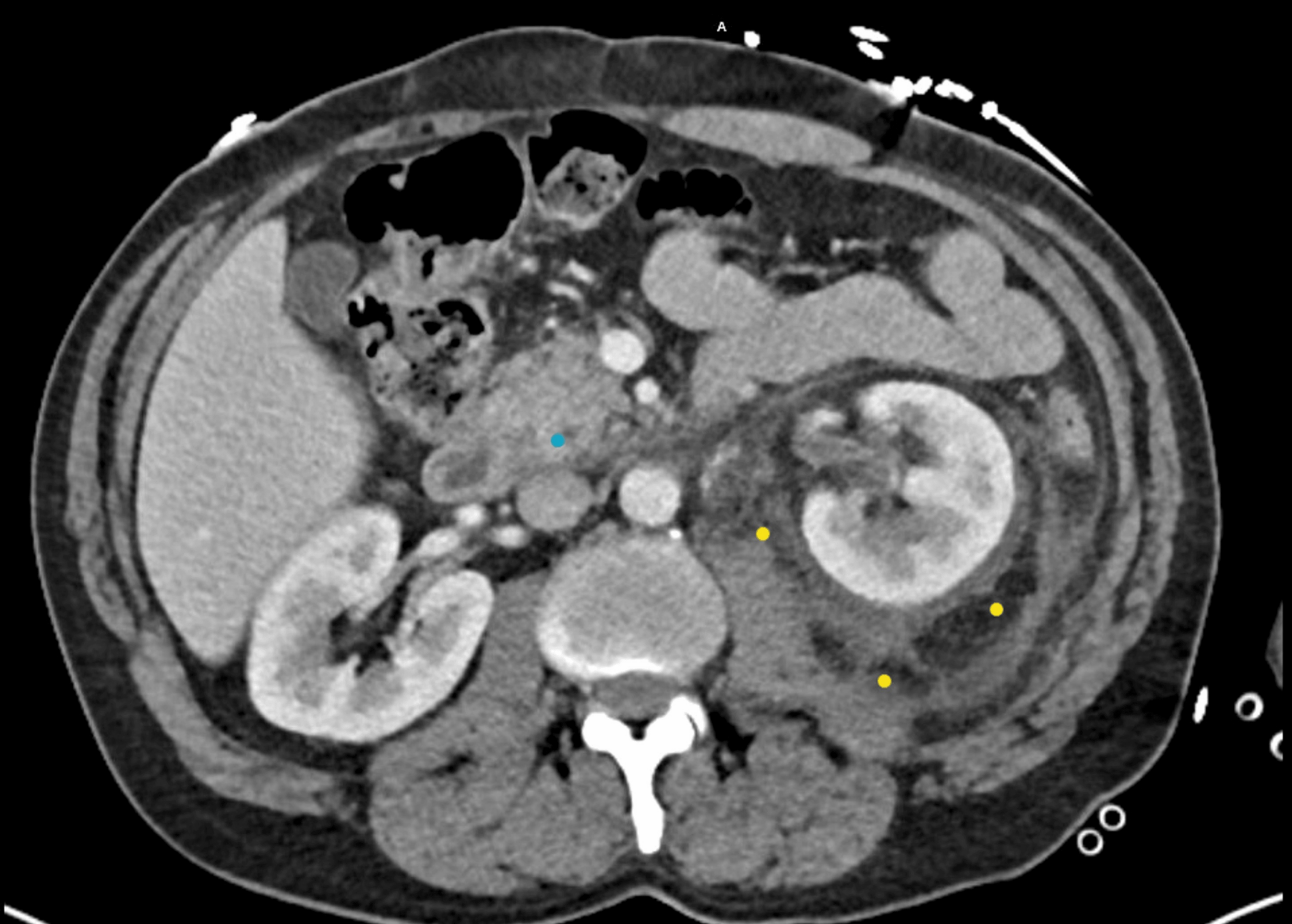
CT Abdomen Showing Perinephric Fluid Collection As Well as Peripancreatic Fluid Collection CT scan of the abdomen showing two areas of fluid collection. The yellow markings highlight the perinephric fluid collection. The blue markings highlight the peripancreatic fluid collection.

**Figure 2:**
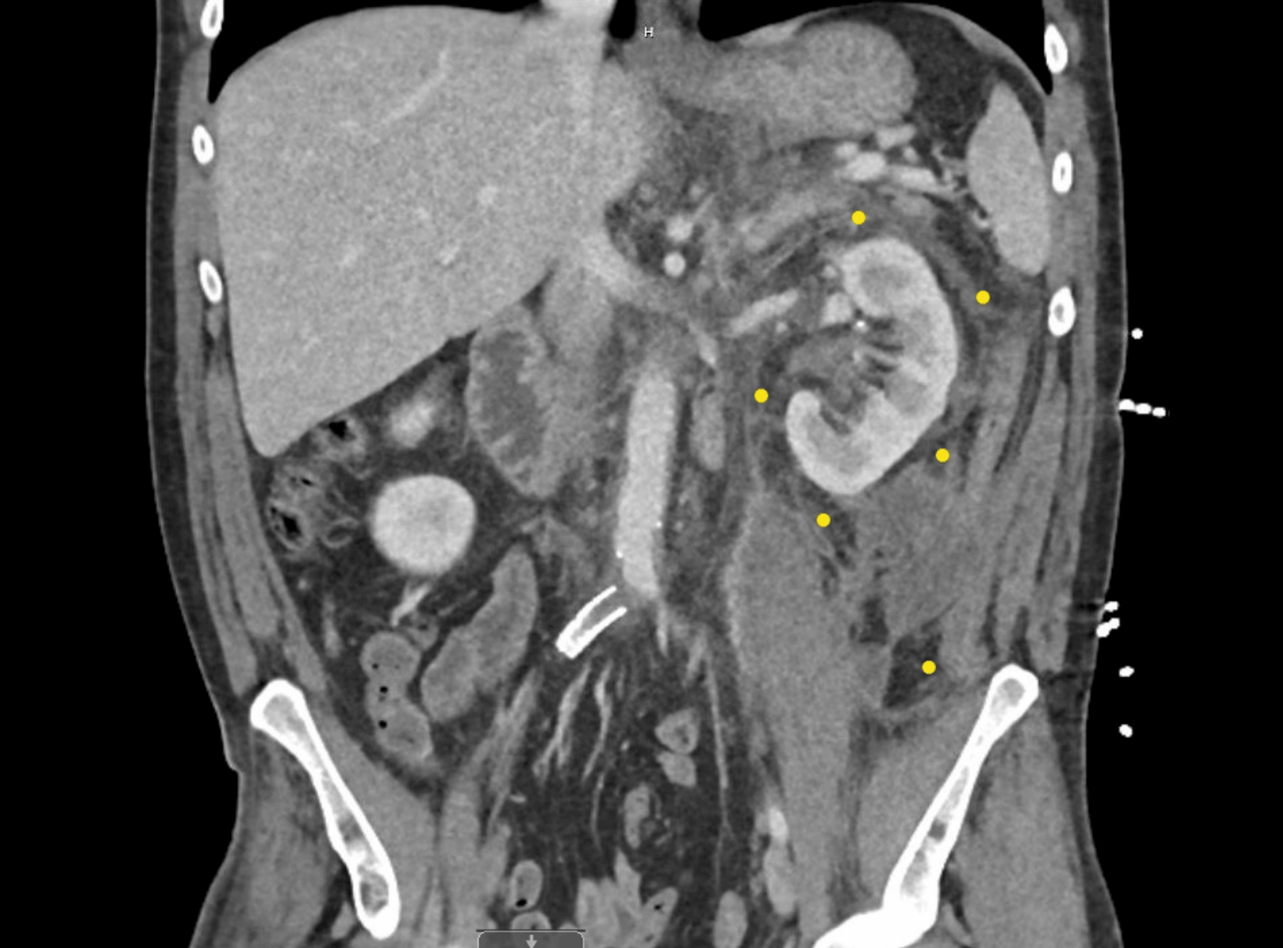
CT Abdomen Redemonstrating the Perinephric Fluid Collection in the Left Perinephric Space CT scan of the abdomen in coronal plane showing extensive fluid collection in the left perinephric space highlighted by yellow markings.

A computed tomography (CT) scan of the abdomen and pelvis with contrast was obtained, which demonstrated severe left peri-nephric stranding with regions of fluid surrounded by peripheral enhancement in the perinephric space in a superomedial direction that measures approximately 6.4 x 2.4 x 4.8 cm, as demonstrated in Figure 1. Additional peripherally enhancing collections extended inferiorly along the medial left intra-abdominal wall enhancing collections extend inferiorly along the medial left intra-abdominal wall toward the inguinal ring, approximately 4.2 x 1.9 x 9.7 cm, as demonstrated in Figure 2. There were mildly prominent left periaortic lymph nodes.

Interventional radiology (IR) was consulted, and the patient’s history, presentation, and emergency department (ED) workup concerning acute pancreatitis were discussed with the on-call IR physician. IR agreed with the initial reading of the CT scan showing multiple small abscesses around the pancreas, spleen, and surrounding the left kidney. IR did not discern that the fluid collections would be amenable to drainage. IR recommended IV antibiotics for a possible infectious cause.

In the ED, the patient was started on the broad-spectrum antibiotics piperacillin-tazobactam and vancomycin. He was given IV (intravenous) fluids, and his pain and nausea were controlled with pain and anti-nausea medications. The patient was admitted to the hospital. After two days, there was an improvement in his leukocytosis with a white blood cell count of 11,000, a decrease in his lipase level to 113 U/L, and there was no growth of his blood and urine cultures. With continued clinical improvement, his IV antibiotics were transitioned to amoxicillin-clavulanic acid, and he was discharged home without sequelae of intra-abdominal abscess. 

## Discussion

The presence of intra-abdominal abscesses in the setting of acute pancreatitis is unusual. Common complications of acute pancreatitis include pancreatic pseudocysts, walled-off necrosis, and disconnected pancreatic duct syndrome [18]. Pancreatic abscess is a rare complication of acute pancreatitis, with an incidence of 1-5% [19], and it is most commonly found in association with acute necrotizing pancreatitis [20]. Intra-abdominal abscesses caused by acute pancreatitis are even more uncommon. Perinephric abscesses have been described as resulting from etiologies such as fistula formation from acute pancreatitis, autoimmune pancreatitis, chronic calcific pancreatitis [21,22], and even pancreatic pseudocysts that mimic a perinephric abscess [23].

When a patient presents with acute pancreatitis and an intra-abdominal abscess, as in this case, it is important to consider whether the intra-abdominal abscess could be the cause of the pancreatitis or, perhaps, they could be unrelated. In this case, the patient presented with a known perinephric abscess that had worsened since a recent drainage at an outside hospital. The fact that the patient did not receive antibiotics post-drainage could have potentiated the infection. Intra-abdominal infections outside the pancreas are not usually considered a common etiology of acute pancreatitis. The worsening of the patient’s perinephric abscess with concomitant acute pancreatitis in the absence of alcohol use and biliary pathology may explain the etiology. However, to our best knowledge, there are no descriptions of such a cause in the literature, and in a patient with a better-described etiology of acute pancreatitis, this would be unlikely.

The patient was admitted and received appropriate care for acute pancreatitis with fluid replacement, pain control, advancement of enteral diet, and clinical/laboratory monitoring. The patient’s perinephric abscess was treated with the broad-spectrum antibiotics vancomycin and piperacillin-tazobactam, which was de-escalated to piperacillin-tazobactam alone. IR was consulted for drainage of his perinephric abscess; however, they did not feel that the abscess was amenable to drainage. In retrospect, it would have been ideal to have cultures from the patient’s percutaneous drainage of the perinephric abscess from the outside facility in case tailoring of antibiotics was necessary. 

The patient’s blood cultures reported no growth after five days, and the urine culture reported no growth after two days. The negative blood cultures showed a lack of systemic infection. The negative urine culture suggests the abscess did not communicate with the kidney’s collecting system, which is not uncommon for a perinephric abscess [24]. 

Acute pancreatitis is an important component of the differential diagnosis of abdominal pain. The chief complaint of abdominal pain accounts for 5-10% of all presentations in the emergency department, and epidemiologic studies reveal acute pancreatitis to be the most common reason for hospital admission in patients with abdominal pain. This case report presents a case of abdominal pain caused by acute pancreatitis, but with an uncommon complication of perinephric abscess. While the vast majority of acute pancreatitis presentations will be without rare complications, such as intra-abdominal abscess, it is essential for clinicians to be conscious of them, as they can present a diagnostic obstacle.

## Conclusions

Pancreatitis is a common condition that can lead to a spectrum of complications, most often involving pseudocysts, necrosis, or infection, but rarely extending into the perinephric space. Awareness of these atypical manifestations is important because they may mimic primary renal pathology.

This report describes a patient who presented with generalized abdominal pain and leukocytosis, who was then found to have a perinephric abscess that was ultimately attributed to acute-on-chronic pancreatitis. The atypical location initially raised concern for a renal infection, underscoring the diagnostic challenge.
